# KPC-mediated resistance in *Klebsiella pneumoniae* in two hospitals in Padua, Italy, June 2009-December 2011: massive spreading of a KPC-3-encoding plasmid and involvement of non-intensive care units

**DOI:** 10.1186/1757-4749-4-7

**Published:** 2012-07-16

**Authors:** Sara N Richter, Ilaria Frasson, Elisa Franchin, Cristina Bergo, Enrico Lavezzo, Luisa Barzon, Antonietta Cavallaro, Giorgio Palù

**Affiliations:** 1 Department of Molecular Medicine, University of Padua, Via Gabelli, 63, 35121, Padua, Italy; 2 Azienda Ospedaliera di Padova, Microbiology and Virology Unit, via Giustiniani 2, 35121, Padua, Italy

**Keywords:** KPC, Carbapenemase, *Klebsiella pneumoniae*, Plasmid-mediated antimicrobial resistance, Gram-negative, Nosocomial infections

## Abstract

**Background:**

*Klebsiella pneumoniae* carbapenemases (KPCs) producing bacteria have emerged as a cause of multidrug-resistant nosocomial infections worldwide. KPCs are plasmid-encoded enzymes capable of hydrolysing a broad spectrum of beta-lactams, including carbapenems and monobactams, therefore worryingly limiting antimicrobial treatment options. Analysis of circulating bacterial strains and KPC alleles may help understanding the route of KPC dissemination and therefore help containing the infection.

**Methods:**

KPC-producing *Klebsiella pneumoniae* dissemination in two 1580- and 300- bed hospitals in Padua, Italy, from initial outbreak in 2009 to late 2011 was analysed. Molecular and clinical epidemiology, including bacterial strains, KPC-encoding plasmid sequences and associated resistance genes, involved hospital wards and relocation of patients were described. Routine antimicrobial susceptibility testing and MIC of carbapenems on clinical isolates were performed. Detection of resistance genes was obtained by PCR and sequencing. MLST, PFGE and ERIC were used for molecular genotyping. Plasmid analysis was obtained by digestion with restriction enzymes and deep sequencing.

**Results:**

KPC-positive clinical samples were isolated from nearly 200 patients. In the initial outbreak intensive care units were almost exclusively involved, while medical, surgical and long-term wards were successively massively concerned. Analysis of KPC alleles, plasmids and bacterial sequence types (STs) indicated that during the initial outbreak KPC-3 in ST258 and KPC-2 in ST147 were each confined in one of the two surveilled hospitals. While KPC-2 dissemination was effectively contained, KPC-3 in ST258 cross-spreading was observed. The simultaneous presence of two carbapenemases, VIM-1 and KPC-2, in the same isolate was also observed in three patients. Total sequencing of plasmid content of two KPC-3 strains showed novel association of resistance plasmids.

**Conclusions:**

The acquired molecular epidemiology demonstrated that 1) both acquisitions from outward sources and patient relocation within the hospitals were responsible for the observed spreading; 2) KPC-3-encoding *Klebsiella pneumoniae* ST258 prevailed over other strains. In addition, the described massive transfer of KPC-mediated resistance to non-intensive care units may anticipate spreading of resistance to the non-hospitalized population. Therefore, genotypic analysis alongside phenotypic identification of carbapenemase producers, also at the carriage state, is advisable to prevent and contain further carbapenemase resistance dissemination.

## Background

Bacteria producing *Klebsiella pneumoniae* carbapenemases (KPCs) have emerged as a cause of multidrug-resistant nosocomial infections worldwide [1]: they are capable of hydrolysing a broad spectrum of beta-lactams including penicillins, cephalosporins, carbapenems and monobactam [2], except cephamycins [3]. The emergence of carbapenem-resistant *Enterobacteriaceae* is particularly worrisome because carbapenems are widely regarded as the drugs of choice for the treatment of severe infections caused by extended-spectrum beta-lactamase (ESBL)-producing *Enterobacteriaceae*[4]; the additional frequent co-occurrence of genes conferring resistance to other classes of drugs has severely limited antimicrobial treatment options [1]. KPC-producing *Klebsiella pneumoniae* isolates were first identified in North Carolina in 1996 [5]; the first outbreak of KPC-producing *K. pneumoniae* outside the United States was described in Israel in 2006 [6]. The European country in which most cases have been reported so far is Greece, where the infection is considered endemic [7]. In Italy a rapid increase in the number of cases has been recently described [8]. However, no long-term surveillance has been reported in Italy, yet. In general, in all countries outbreaks have always been restricted to hospitals, in particular to intensive care units (ICU) [9].

KPCs are plasmid-encoded enzymes whose high mobility and dissemination are related to a Tn3-based transposon, Tn*4401*, which is carried by large plasmids varying in size and structure [1]. The most frequently found KPC-encoding plasmid is pKpQIL [10]. To date 11 different KPC variants (KPC1/2-KPC12) have been described [11]. In Italy both KPC-2 and KPC-3 have been reported, with a higher prevalence of KPC-3. Many sequence types (STs) of *K. pneumoniae* have been found associated to KPC enzyme production: the most globally distributed, therefore likely the most environmentally fit, is ST258, which was found coupled to both KPC-3 and KPC-2 [12].

Here we present the first long-time follow up of KPC dissemination in Italy. KPC clinical isolates were surveilled from initial outbreak in 2009 until late 2011 in two hospitals (nearly 2000 beds) in Padua, a large town in North-East Italy counting over 936.000 inhabitants, including provincial citizens. In particular, we focussed on KPC allele dissemination, *K. pneumoniae* ST spreading and identity of KPC-encoding plasmids with respect to the two involved hospitals. In addition, the clinical epidemiology showed worrying progressive transfer of KPC infection from intensive care units (ICUs) to surgical, medical and long-term hospital wards.

## Results

Starting in 2009, standard microbiological testing revealed a dramatic rise in the number of *K. pneumoniae* isolates with increased MIC of imipenem (≥2 mg/L) in the intensive-care units (ICU) of the Teaching Hospital (TH, one ward) and Saint Antony Hospital (SAH, one ward), two distinct hospital complexes in the city of Padua, offering 1580 and 300 beds, respectively. In particular, in ICU/TH and ICU/SAH, isolates with imipenem MIC ≥2 mg/L were absent until June 2009, increased from 17% and 12%, respectively, in July-December 2009, to 87% and 54%, respectively, in January-June 2010. From June 2009, we started to search for KPC genes on clinical isolates exhibiting imipenem MIC ≥2 mg/L, within all units of the two hospitals. In addition, since summer 2010 a systematic search for KPC-positive isolates was performed twice a week by rectal swabbing of all patients from ICU/TH.

### Molecular and clinical epidemiology

**Figure 1:**
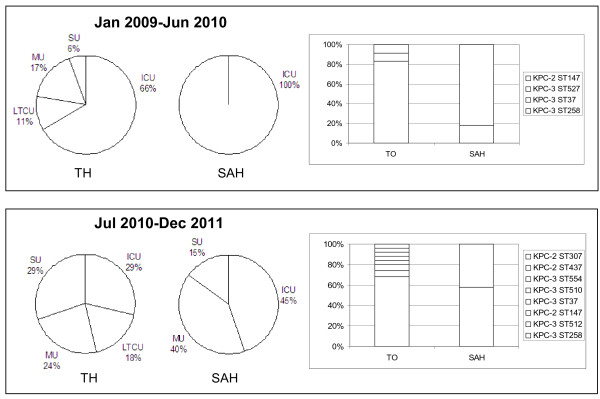
**Epidemiology of KPC genes in two hospitals (TH and SAH).** On the left, pie charts show the evolution of KPC spreading in hospital wards from January 2009 to Jun 2010 and from July 2010 to December 2011. On the right, bar graphs indicate prevalence of KPC genes and their association with *K. pneumoniae* STs during the two considered periods of time. Abbreviations are: ICU = Intensive Care Unit; MU = Medical Unit; SU = Surgical Unit; LTCU = Long Term Care Unit; TH = Teaching Hospital; SAH = Saint Antony Hospital.

**Figure 2:**
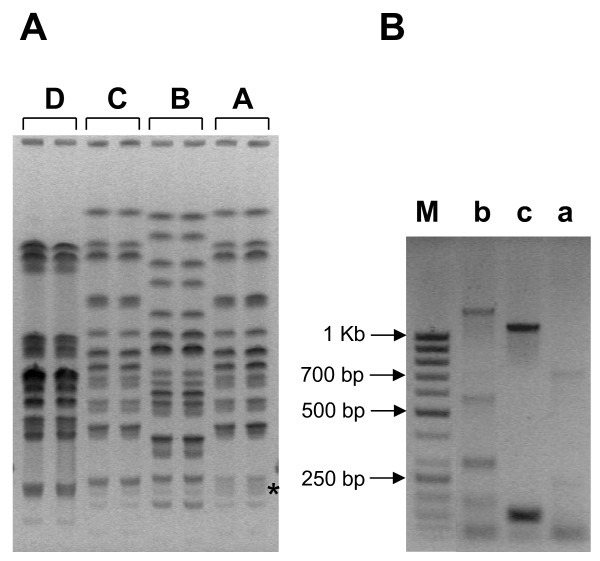
**Bacterial typing of*K. pneumoniae*clinical isolates.A)** PFGE of representative samples belonging to different STs as measured by MLST. Samples were arbitrarily described by capital letters which designate their PFGE profile. Each sample was run in duplicate. The asterisk indicates the region where profile A differs from profile C. A, B, C and D PFGE profiles corresponded to ST147, ST258, ST527 and ST37, respectively. **B)** Gel electrophoresis of ERIC-PCR of representative clinical isolates. Samples were arbitrarily described by lowercase letters which designate their ERIC profile. ERIC profiles a, b and c corresponded to ST147, ST258 and ST527 (see also Table 1).

Up to June 2010, 29 patients with at least one KPC-positive *K. pneumoniae* isolate were identified: as shown in Figure 1, in both hospitals the vast majority (66% and 100%) was from ICUs. Remarkably, two different KPC genes in two *K. pneumoniae* sequence types (STs) were prevalent in the two hospitals: in particular, KPC-3 in ST258 (83%) had spread in TH, while KPC-2 in ST147 (82%) was prevalently found in SAH. KPC-3-positive isolates belonging to the two new ST527 and ST37 were also detected in TH. Since MSLT takes into account modifications in highly conserved genes hence revealing mainly long-term changes, two additional molecular typing techniques, ERIC-PCR and PFGE, were used to detect short-time alterations. In particular, ERIC-PCR identifies changes in variable regions and PFGE in the whole genome. All clinical isolates were classified into 4 PFGE profiles, represented in Figure 2a, which matched the classification made by MLST. Three profiles were identified with ERIC-PCR (Figure 2b): these matched the MLST and PFGE but for one sample (#7), indicating a less discriminatory power of this technique or possibly a closer genetic relatedness between this sample (encoding KPC-3 in ST37 with PFGE profile D) and samples encoding KPC-3 in ST258 with PFGE profile B. Overall these data indicate that KPC-positive samples derived from four different *K. pneumoniae* strains with poor genetic relatedness. Genotypic and phenotypic properties of these KPC-positive isolates are reported in Table 1.

**Table 1:** Properties of KPC-positive clinical isolates and transformant strains. Isolates were collected from 29 patients in the time period January 2009-June 2010

**Sample ID**	**Hospital unit**	**carbapenemase**		**β-lactamase**				**molecular typing**			**MIC (mg/L)**								
		**KPC**	**VIM**	**TEM**	**SHV**	**CTX**	**OXA**	**MLST**	**PFGE**	**ERIC**	**IPM**	**MER**	**CTX**	**CAZ**	**FEP**	**SXT**	**GEN**	**AMK**	**TGC**
**13**	SAH-ICU	2	1	1	12	nd	9	147	A	a	≥16	≥16	≥64	≥64	≥64	≥320	4	32	2
**20**	SAH-ICU	2	1	1	12	nd	9	147	A	a	≥16	≥16	≥64	≥64	≥64	≥320	4	≥64	2
**39**	SAH-ICU	2	nd	1	nd	nd	9	147	A	a	≥16	≥16	≥64	≥64	≥64	40	≤1	≤2	2
**40**	SAH-ICU	2	nd	1	nd	nd	9	147	A	a	≥16	≥16	32	16	32	40	≤1	≤2	2
**Top10-41**											8, *2*	2, *0.5*	≥64	4	2	≤20	≤1	≤2	≤0.5
**42**	SAH-ICU	2	nd	1	12	nd	9	147	A	a	≥16	≥16	32	16	32	40	≤1	≤2	2
**46**	SAH-ICU	2	nd	1	12	nd	9	147	A	a	≥16	≥16	32	16	≥64	40	≤1	≤2	2
**49**	SAH-ICU	2	1	nd	nd	nd	9	147	A	a	≥16	≥16	32	16	≥64	40	≤1	≤2	2
**65**	SAH-ICU	3	nd	1	11	nd	9	258	B	b	≥16	4	8	≥64	8	≥320	2	≥64	2
**68**	SAH-ICU	3	nd	1	11	nd	9	258	B	b	≥16	4	32	≥64	8	≥320	4	≥64	2
**69**	SAH-ICU	2	nd	1	12	nd	9	147	A	a	≥16	≥16	32	16	≥64	40	≤1	≤2	2
**Top10-69**											8, *3*	1, *1*	≥64	4	2	≤20	≤1	≤2	≤0.5
**74**	SAH-ICU	2	nd	nd	nd	nd	nd	147	A	a	≥16	≥16	≥64	≥64	≥64	≥320	4	≥64	2
**35**	TH-ICU	3	nd	1	nd	nd	9	258	B	b	≥16	8	≥64	≥64	16	≥320	≥16	8	2
**Top10-35**											8, *2*	1, *0.75*	≥64	4	2	≤20	≤1	≤2	≤0.5
**44**	TH-ICU	3	nd	1	nd	nd	9	258	B	b	8	8	8	≥64	16	≥320	≥16	16	2
**Top10-44**											8, *1.5*	1, *0.5*	≥64	4	2	≤20	≤1	≤2	≤0.5
**45**	TH-ICU	3	nd	1	11	nd	9	258	B	b	≥16	2	≥64	≥64	16	≥320	≥16	8	2
**48**	TH-ICU	3	nd	nd	nd	nd	9	258	B	b	≥16	8	≥64	≥64	16	≥320	≥16	8	2
**53**	TH-ICU	3	nd	1	nd	nd	9	258	B	b	≥16	8	≥64	≥64	16	≥320	≥16	≥64	2
**Top10-53**											8, *2*	2, *0.38*	≥64	4	2	≤20	≤1	≤2	≤0.5
**63**	TH-ICU	3	nd	1	nd	nd	9	258	B	b	≥16	8	≥64	≥64	32	≥320	≥16	16	2
**71**	TH-ICU	3	nd	1	11	nd	9	258	B	b	4	2	16	≥64	8	≥320	≥16	8	2
**72**	TH-ICU	3	nd	1	11	nd	9	258	B	b	≥16	4	16	≥64	8	≥320	4	≥64	4
**12**	TH-SU		nd	nd	nd	nd	nd	527	C	c	≥16	≤0.25	≤1	≤1	≤1	≥320	≤1	4	2
**Top10-12**											8, *2*	2, *0.5*	≥64	4	2	≤20	≤1	≤2	≤0.5
**14**	TH-ICU	3	nd	1	11	nd	9	258	B	b	≥16	8	8	≥64	8	≥320	2	≥64	4
**19**	TH-ICU	3	nd	1	11	nd	9	258	B	b	≥16	2	8	≥64	≥64	≥320	2	≥64	2
**8**	TH-MU	3	nd	1	11	nd	9	258	B	b	≥16	2	8	≥64	16	≥320	4	≥64	2
**17**	TH-MU	3	nd	1	11	nd	9	258	B	b	≥16	4	8	≥64	8	≥320	4	≥64	4
**18**	TH-MU	3	nd	1	11	nd	9	258	B	b	4	2	16	≥64	8	≥320	4	≥64	4
**4**	TH-LTCU	3	nd	1	11	nd	9	258	B	b	4	2	8	≥64	8	≥320	4	≥64	2
**7**	TH-LTCU	3	nd	1	11	15	9	37	D	b	≥4	2	≥64	≥64	≥64	≥320	8	≤2	2
**Top10-7**											8, *3*	2, *1*	≥64	4	2	≤20	≤1	≤2	≤0.5
**21**	TH-ICU	3	nd	1	11	nd	9	258	B	b	≥16	≥16	≥64	≥64	≥64	≥320	4	≥64	1
**64**	TH-ICU	3	nd	1	nd	nd	9	258	B	b	≥16	8	8	≥64	16	≥320	≥16	16	2
**Top10-64**											8, *2*	1, *0.38*	≥64	4	2	≤20	≤1	≤2	≤0.5
**Top10**											≤1, *0.25*	≤0.25, *0.32*	≤1	≤1	≤1	≤20	≤1	≤2	≤0.5

IPM = imipenem; MER = meropenem; CTX = cefotaxime; CAZ = ceftazidime; FEP = cefepime; SXT = sulfamethoxazole-trimethoprim; GEN = gentamicin; AMK = amikacin; TGC = tigecycline. Transformed *E. coli* strains are indicated as Top10 and the number corresponding to the clinical sample from which the plasmid was obtained.

In transformed Top10 strains, MIC values of imipenem and meropenem were obtained by Vitek (first MIC value) and E-test (second MIC value in italics).

Carbapenem resistance coupled to KPC production was followed up from July 2010 to December 2011. A total of 160 different patients with at least one isolate were found. Of these, 140 patients were in TH. Remarkably, in this time period in both hospitals KPC-positive isolates were mainly detected in non-ICUs, such as surgical, medical and long-term care wards (Figure 1).

Molecular analysis showed that in the second period of analysis both KPC-2 and KPC-3 had moved within the two hospitals: in particular, KPC-3-positive isolates belonging to ST258 were mostly represented in both nosocomia (Figure 1). Concurrently, several novel STs, such as ST512, ST745 (two new single locus variants of ST258, in *gapA* and *rpoB,* respectively), ST510, ST554 associated with KPC-3, and ST307, ST437 with KPC-2 were detected.

**Figure 3:**
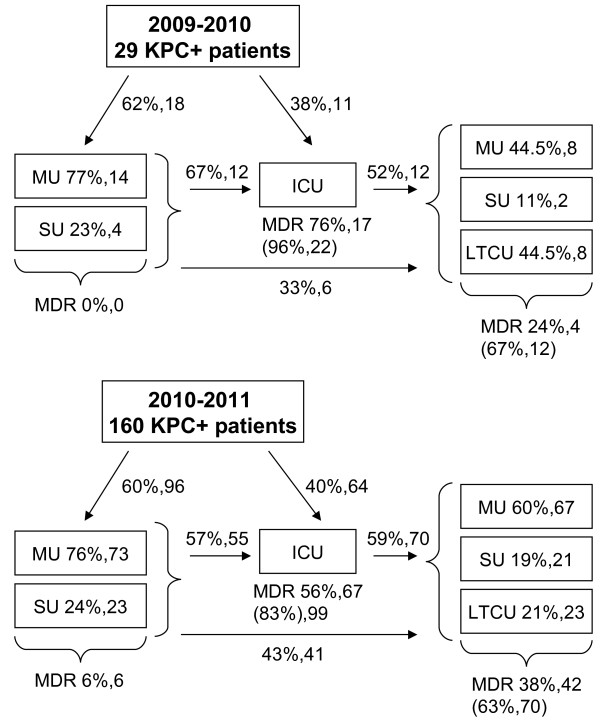
**Relocation of KPC-positive patients among different hospital wards.** Relocation has been analyzed in the two periods of time: Jan 2009-Jun 2010 and Jul 2010-Dec 2011. Percentage of MDR isolates (including KPC-positive strains) were calculated on the total number of KPC-positive patients (29 and 160 for the first and second period of analysis, respectively). The first number describes MDR percentages of patients that newly acquired MDR organisms in the indicated hospital units; the second number refers to the absolute number of patients. The third bracketed number describes the percentage of patients found with MDR isolates (both newly and previously acquired) over the total number of KPC-positive patients hospitalized in the indicated ward. Abbreviations are: ICU = Intensive Care Unit; MU = Medical Unit; SU = Surgical Unit; LTCU = Long Term Care Unit; MDR = multi-drug-resistance.

To understand the involvement of non-ICUs after the initial outbreak, relocation of patients was studied in the two periods of time (Jan 2009-Jun 2010 and Jul 2010-Dec 2011) (Figure 3). In the first period even though most of the patients were initially admitted to non-ICUs, the vast majority of multi-drug-resistant (MDR) isolates, including KPC-positive bacteria, were found during the following ICU admission (76%) and were mostly maintained in subsequent post-ICUs, where the percentage of new acquisitions was 24%. In the second period, the number of new acquisitions of MDR organisms (MDRO) dramatically raised in non-ICUs (6% and 38% in pre-ICU and post-ICU admission, respectively), while new acquisitions in ICU decreased. Noteworthy, the percentage of patients with newly and already acquired MDR isolates was very high with respect to the total number of KPC-positive patients hospitalized in ICUs, both in the first and second period (96% and 83%, respectively), and was in general maintained in post-ICUs (67% and 63%, respectively).

To slow down KPC spread, starting from 2011, one ICU has been devoted to patients colonized/infected by KPC organisms. In addition, most non-ICUs introduced the policy of isolating KPC-positive patients in a dedicated room, with restricted access to hospital staff and relatives.

### Plasmid analysis

**Figure 4:**
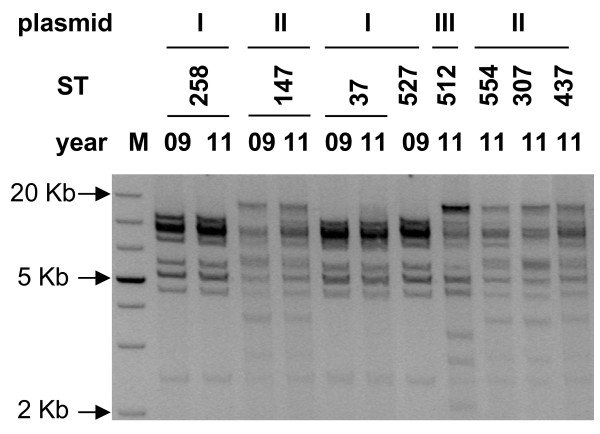
**Digestion profiles of purified KPC-encoding plasmids: different digestion patterns, classified as I, II and III, are shown on the top of the figure.** STs and year of isolation are shown above the gel. M stands for markers of molecular weights.

To verify the similarity of KPC-positive plasmids, plasmids from representative samples from each identified ST were transformed into *E. coli* Top10, purified, digested with restriction enzymes and run on agarose gel. Additionally, plasmids from ST258, ST147 and ST37 detected in November 2009 were compared to those collected in September 2011 to check for plasmid variation within the same ST. As shown in Figure 4, no variation was found between plasmids collected in 2009 and 2011 (see ST258, ST37, ST147). In addition, KPC-3-encoding plasmids from ST258, ST37 and ST527 displayed identical digestion profiles, but differed from that from ST512. KPC-2-positive plasmids from ST147, ST307 and ST437 exhibited a unique digestion pattern.

Partial plasmid sequencing revealed that *bla*_KPC_ genes were in all cases embedded in a Tn4410-like transposon. The KPC-3-encoding plasmids present in *K. pneumoniae* ST258 (identical to the plasmid contained in ST37 and ST147) and ST512 were further subjected to deep sequencing. We found that both KPC-3 plasmid sequences matched that of pKpQIL, which has been previously reported in *K. pneumoniae* ST258 [10]. *K. pneumoniae* ST512 additionally contained a second plasmid identical to the recently described plasmid pIncX-SHV [13], which explains the above depicted partially different digestion profile.

### Phenotypic and molecular analysis of additional resistance determinants

The presence of KPC-encoding plasmids increased the MIC of imipenem and meropenem from 0.25 and 0.32 mg/L, respectively, to 1.5-3 and 0.38-1 mg/L, respectively. In addition, transformed Top10 cells gained resistance to β-lactams, due to *bla*_TEM-1_ and *bla*_OXA-9_ genes present in all transformed plasmids, while remained susceptible to sulfamethoxazole/trimethoprim, aminoglycosides and tigecycline, indicating that KPC-plasmids did not contain additional resistance genes to these classes of antibiotics.

Other chromosomal resistance genes or genes located on different plasmids were present in the clinical isolates. Antibiotic susceptibility testing showed that isolates were multiresistant and retained susceptibility only to tigecycline and colistin. Molecular analysis showed the presence of *bla*_SHV-12_ (58%) and *bla*_SHV-11_ (50%) in KPC-2 and KPC-3-positive strains, respectively. Noteworthy, the latter association (i.e KPC-3 and *bla*_SHV-11_) has only been reported in Northern Europe and USA [14,15]. Most of the isolates were positive for *bla*_TEM-1_ (86%), *bla*_CTX-M-15_ (2.5%), and *bla*_OXA-9_ (88%). Interestingly, 4 KPC-2 isolates were additionally positive for the metallo-β-lactamase *bla*_VIM-1_, association which has been reported only in Greece and very recently in Germany [16,17]. In our clinical setting, there was no evidence of extensive spreading of *bla*_VIM-1_ in other species.

## Discussion and conclusions

Analysis of KPC-mediated resistance in two hospitals in Padua, Italy, revealed two distinct spreading behaviours: during initial outbreak, ICUs were almost exclusively involved. Each of the two identified *bla*_KPC_ genes identified was very clearly confined in only one of the two hospitals. Four *K. pneumoniae* STs were identified: ST258 associated with both KPC-2 and KPC-3 has been reported worldwide [1], and in particular in Italy [8]; ST147 associated with KPC-2 has been detected in Greece [7], while ST37 and ST527 have never been identified associated with a bla_KPC_ gene. These 4 STs differed for at least 5/7 loci, hence had poor genetic relatedness, indicating nosocomial acquisition of *bla*_KPC_ both by outward sources (e.g. patients that travelled abroad or were moved from other hospitals) and by horizontal transfer of KPC-encoding plasmids. KPC spreading was additionally favoured by clonal expansion of KPC-positive ST258 and ST147, as attested by prevalence of these STs in each of the two hospitals.

A strikingly different behaviour occurred in the second period of analysis: KPC-mediated resistance massively moved from ICUs to medical, surgical and long-term care wards. Additionally, KPC-3-positive ST258 prevailed over KPC-2 ST147, and many novel STs, which have never been reported associated with KPC, were found. One KPC-3-encoding plasmid, pKpQIL [10], was detected in most of the isolates, indicating that this plasmid backbone has superior conjugation/mobility properties with respect to other identified plasmids.

In conclusion, our work showed that KPC genes have different mobility ability, some of them being very efficient in horizontal transfer within *K. pneumoniae* strains. The important consequence is that KPC-mediated resistance is now present also in non-ICUs, with subsequent dangerous possible spreading to the community. This behaviour, even though detected in an Italian city, very likely reflects KPC spreading evolution worldwide. Both phenotypic and genotypic identification of carbapenemase producers, also at the carriage state, is thus urgently needed to prevent and contain further resistance spreading, especially in the case of highly transmissible plasmids.

## Methods

### KPC outbreak investigation and *follow-up*

In the city of Padua, in North-East Italy, the two main public hospitals are the Teaching Hospital (TH) and the Saint Antony Hospital (SAH), which offer 1580 and 300 beds, respectively. They are two distinct and independent hospital complexes, located around 1.5 km apart. Both offer clinical support in all areas of medicine. There is no routine relocation of patients, nor staff exchange between the two hospitals. Phenotypic and genotypic KPC investigation was performed from June 2009 to December 2011. Investigated clinical specimens were: blood, urine, bronchoalveolar lavage, sputum, perirectal swab, nasal swab, wound swab, skin swab, and liquor.

### Clinical epidemiology

Hospitalization data from June 2009 to December 2011 were analyzed for each patient. Considering that only few patients had been screened for KPC by genotypic methods since the beginning of their hospitalization, we assumed that KPC was acquired when the first MDR isolate (including resistance to imipenem and meropenem) was phenotypically detected. We considered this a reliable assumption because we had observed that all *K. pneumoniae* phenotypically resistant to imipenem and meropenem encoded the KPC enzyme. Moreover, patients were considered to have been newly infected/colonized with MDR organisms when at least two samples obtained from the same unit and from the same type of clinical sample had been previously found negative. Patients with at least one KPC-positive sample in a different ward or in a different type of sample were considered to have been previously infected/colonized, so that for each patient no more than one new acquisition was counted. Definitions: “KPC-positive *K. pneumoniae*” refers to strains that were genotypically confirmed to encode the KPC enzyme; “carbapenem-resistance” indicates resistance to carbapenem antibiotics and may be encoded by KPC or other carbapenemases; “multi-drug-resistant (MDR) organism” refers to bacteria that were phenotypically found to be resistant to all major classes of antibiotics, including carbapenems.

### Antimicrobial susceptibility testing and carbapenemase screening

Clinical specimens were streaked on non-selective enrichment agar plates (BBL Blood Agar or BBL Chocolate II Agar), and on differential and selective MacConkey II Agar plates (all from Becton Dickinson Italia, Milan, Italy), incubated at 35 °C ± 2 for 16-18 h; if necessary, single colonies (usually, one ore two) of each colony type were further streaked on enrichment agar plates to obtain pure cultures. Microbial identification and antibiotic susceptibility testing was performed using a Vitek2 automated system (bioMerieux, Marcy l’Etoile, France). Resistance was defined using EUCAST criteria (http://www.eucast.org/fileadmin/src/media/PDFs/EUCAST_files/Breakpoint_tables/Breakpoint_table_v_2.0_120221.pdf). The MICs of imipenem and meropenem were determined by the Etest (AB Biodisk, Solna, Sweden). The modified Hodge test was performed to detect carbapenemase production [18].

### PCR for detection of antibiotic resistance genes

Resistance genes (*bla*_KPC_*bla*_TEM_*bla*_SHV_*bla*_CTX-M_*bla*_VIM_*bla*_IMP_*bla*_NMC-IMI_*bla*_SME_*bla*_SPM_*bla*_OXA-1_*bla*_OXA-9_*bla*_OXA-48_*bla*_OXA-58_*bla*_OXA-23_*bla*_OXA-24_*bla*_OXA-51_*bla*_OXA-143_) were detected by PCR and sequencing [19,20]. Positive PCR products were sequenced with an ABI3730 sequencer (Applied Biosystems) and sequences compared with those from GenBank (http://www.ncbi.nlm.nih.gov/blast/).

### Molecular genotyping

The genetic relatedness of carbapenem-resistant strains was determined by multilocus sequence typing (MLST), pulsed-field gel electrophoresis (PFGE) analysis and enterobacterial repetitive intergenic consensus-PCR (ERIC-PCR) [21,22]. MLST was performed according to the MLST website (http://www.pasteur.fr/recherche/genopole/PF8/mlst/Kpneumoniae.html). For PFGE, total DNA extracted from bacterial cells was digested with XbaI (Fermentas Life Science), run on agarose gel with CHEF-DR® III Variable Angle System (Bio-Rad Laboratories, Milan, Italy) and stained with ethidium bromide. PFGE profiles were interpreted according to Tenover criteria [23]. Isolates with the same pulsotype were classified as a clone. ERIC-PCR was performed using reported primers ERIC1 and ERIC2 [20].

### Plasmid analysis and transformation

Plasmid DNA was purified using phenol/chloroform extraction and electroporated (Bio-Rad Gene Pulser®, Bio-Rad Laboratories, Milan, Italy) into *E. coli* Top10 (Invitrogen Ltd., Paisley, United Kingdom) recipient cells. Transformed strains were selected on ampicillin agar plates (100 mg/L) and *bla*_KPC_ presence was confirmed by PCR. Purified plasmids from transformed KPC-positive colonies were digested with EcoRI and XbaI (Fermentas Life Sciences, Milan, Italy) and run on 1% agarose gels to compare their restriction pattern. Transformants possessing *bla*_KPC_ were subjected to antibiotic susceptibility testing and further molecular characterization.

### Plasmid sequencing

Plasmid DNA obtained from transformed bacterial cells was sequenced by sequence walking and cloning of DNA fragments obtained by nebulization (Clonejet kit Fermentas Life Science). Nucleotide sequences were compared to the ones on the NCBI (National Center for Biotechnology Information) website. For deep sequencing, plasmid DNA extracted from samples was sequenced using a 454 FLX Sequencing Platform (Roche). Briefly, purified DNA was fragmented by nebulization, fragments in the size range of ~600-900 base pairs (bp) were selected and adaptor sequences, specific for 454 shotgun sequencing protocol, were ligated to both fragments ends. Genome libraries prepared from each isolate were uniquely bar-coded to allow multiplexing and subsequent in silico deconvolution. We obtained a total of 103914 reads from a pool of 8 samples, with a mean length of 436 bp. Assembly of sequences was conducted with Newbler 2.6 (Roche).

## Abbreviations

KPC, Klebsiella pneumoniae carbapenemase; TH, Teaching Hospital; SAH, Saint Antony Hospital; ICU, Intensive care unit; ST, Sequence type; MLST, multi locus sequence typing; PFGE, pulse field gel electrophoresis; ERIC, Enterobcterial Repetitive Intergenic Consensus; MIC, minimum inhibitory concentration; MDR, multi drug resistance.

## Competing interests

The authors declare no competing interests.

## Author’s contributions

SNR conceived of the study, participated in its design and coordination and drafted the manuscript; IF carried out the genotypic assays; EF carried out the MLST analysis and participated in the deep sequencing analysis; CB participated in the phenotypic analysis; EL carried out the deep sequencing data analysis; LB participated in the deep sequencing analysis; AC conceived of the study and participated in its design and coordination; GP participated in the study coordination. All authors read and approved the final manuscript.
